# Ultrasound and histopathological assessment of benign, borderline, and malignant thyroid tumors in pediatric patients: an illustrative review and literature overview

**DOI:** 10.3389/fendo.2024.1481804

**Published:** 2025-01-30

**Authors:** Dominika Januś, Monika Kujdowicz, Aleksandra Kiszka-Wiłkojć, Konrad Kaleta, Anna Taczanowska-Niemczuk, Jan Radliński, Kamil Możdżeń, Zuzanna Nowak, Wojciech Górecki, Jerzy B. Starzyk

**Affiliations:** ^1^ Department of Pediatric and Adolescent Endocrinology, Chair of Pediatrics, Institute of Pediatrics, Jagiellonian University Medical College, Krakow, Poland; ^2^ Department of Pediatric and Adolescent Endocrinology, University Children Hospital in Krakow, Krakow, Poland; ^3^ Department of Pathomorphology, Jagiellonian University Medical College, Krakow, Poland; ^4^ Department of Pathology, University Children Hospital in Krakow, Krakow, Poland; ^5^ Department of Pediatric Surgery, Institute of Pediatrics, Jagiellonian University Medical College, Krakow, Poland; ^6^ Department of Pediatric Surgery, University Children Hospital in Krakow, Krakow, Poland; ^7^ Department of Pediatric and Adolescent Endocrinology, Chair of Pediatrics, Institute of Pediatrics, Students` Scientific Society, Jagiellonian University Medical College, Krakow, Poland; ^8^ Szpital Zakonu Bonifratrow sw. Jana Grande, Krakow, Poland

**Keywords:** NIFTP, WDT-UMP, FT-UMP, papillary thyroid carcinoma, follicular thyroid carcinoma

## Abstract

**Background:**

The risk of malignancy in thyroid nodules is higher in children than in adults, often necessitating a more aggressive endocrine and surgical approach. However, given that not all solid thyroid nodules are malignant, a more conservative approach may also be appropriate in certain cases.

**Objective:**

This study aims to present an illustrative analysis of the pathological foundations underlying the sonographic appearance of benign, borderline, and malignant thyroid nodules in the pediatric population at a single tertiary thyroid center.

**Methods:**

A total of 47 well-documented pediatric patients referred for thyroid surgery between 2010 and 2023 were analyzed. This retrospective assessment included an examination of demographic data, hormonal profiles, ultrasound findings, and histopathology reports.

**Results:**

Ultrasound and histopathology of thyroid nodules provided insights into subgroup differentiation. Benign nodules like dyshormonogenetic goiter showed solid hypoechoic features on ultrasound and dense fibrosis on histopathology, while thyroid follicular nodular disease exhibited isoechoic nodules with halos, histologically revealing dilated follicles. In borderline tumors, well-differentiated tumor of uncertain malignant potential (WDT-UMP) nodules were hypo/hyperechoic with occasional capsular invasion, resembling papillary thyroid carcinoma (PTC) features histologically. Non-invasive follicular thyroid neoplasm with papillary-like nuclear features (NIFTP) appeared as well-defined hypoechoic nodules with a hypoechoic rim, with histology showing follicular architecture and PTC nuclear features, but no invasion. Follicular tumor of uncertain malignant potential (FT-UMP) displayed hypo/hyperechoic patterns and indistinct borders, with uncertain capsular invasion and no PTC nuclear features. Malignant lesions showed distinct patterns: PTC as hypoechoic, irregular nodules with mixed vascularization, follicular thyroid carcinoma as large, hyperechoic nodules with invasive features, and poorly differentiated thyroid carcinoma (PDTC) as heterogeneous hypoechoic masses.

**Conclusion:**

Because of the significant overlap in sonographic features among benign, borderline, and certain malignant thyroid lesions in pediatric patients, ultrasonography alone is insufficient for accurate risk stratification. This overlap necessitates referrals for fine-needle aspiration biopsy (FNAB) in children more frequently than in adults. Future studies utilizing artificial intelligence (AI) to predict clinical outcomes in thyroid nodule diagnostics may offer new advancements, particularly given the increasing number of pediatric patients with solid thyroid lesions.

## 1 Introduction

Ultrasound (US) imaging plays a vital role in evaluating thyroid nodules, especially following the identification of low-risk (borderline) neoplasms, such as non-invasive follicular thyroid neoplasm with papillary-like nuclear features (NIFTP) and thyroid tumors of uncertain malignant potential: follicular tumor of uncertain malignant potential (FT-UMP) and well-differentiated tumor of uncertain malignant potential (WDT-UMP) (1, 2).

Epidemiological studies have shown a global increase in the prevalence of thyroid nodules, with rates among adults ranging from 33% to 68% (3, 4). In the general pediatric population, the incidence of thyroid nodules is lower (0.5% to 2%) compared to adults, though it is higher (3.5% to 31.5%) in children with autoimmune thyroiditis (AIT) (5–8). Pediatric thyroid nodules carry a 9.2%–50% risk of malignancy (ROM), compared to 5% to 15% in adults (9, 10). A 2023 study by Huang et al. noted an increasing incidence of thyroid cancer (TC) in individuals under 40 years of age in several countries, including Poland (11).

Given the higher malignancy risk in pediatric thyroid nodules compared to adults, treatment tends to be more aggressive (9, 10, 12, 13). However, not all solid thyroid nodules in children are malignant, suggesting that a more conservative approach could be appropriate in some cases (13). A prime example is the reclassification of encapsulated follicular variant of papillary thyroid carcinoma (EFVPTC) as NIFTP, which has significantly altered the therapeutic approach—from total thyroidectomy with radioiodine therapy to lobectomy with potential follow-up (14, 15).

For clinical pediatric endocrinologists, the updated 2022 World Health Organization (WHO) Classification of Thyroid Tumors has been particularly significant, highlighting the role of thyroid pathologists in decision-making (2). Most thyroid tumors originate from follicular epithelial cells and are categorized into benign, low-risk (borderline), and malignant neoplasms (2). Benign tumors include thyroid follicular nodular disease (TFND), follicular thyroid adenoma, follicular thyroid adenoma with papillary architecture, and oncocytic adenoma (OCA) of the thyroid (2). Low-risk neoplasms include NIFTP, FT-UMP, WDT-UMP, and hyalinizing trabecular tumor (2). Malignant neoplasms include follicular thyroid carcinoma (FTC), invasive EFVPTC, papillary thyroid carcinoma (PTC), oncocytic carcinoma of the thyroid, follicular-derived carcinomas, high-grade [poorly differentiated thyroid carcinoma (PDTC), differentiated high-grade thyroid carcinoma], and anaplastic follicular cell-derived thyroid carcinoma (2).

The presentation of TC, particularly PTC, in children is typically more severe than in adults, leading to more extensive surgical interventions, including total thyroidectomy, lymphadenectomy, and 131I therapy, which may result in significant long-term side effects (9, 10, 12, 13, 16). Therefore, ongoing research in the pediatric population should focus on improving the visualization of thyroid nodules and refining histopathological assessments to minimize the side effects of aggressive surgical approaches in children with benign and borderline tumors, who have a long life expectancy (2, 13, 16, 17).

Since the introduction of NIFTP in 2016 and the publication of the fifth edition of the WHO Classification of Thyroid Tumors in 2022, our center has revised pediatric histopathological assessments, leading to the diagnosis of 18 borderline tumors (2, 14, 15).

Sonographic assessment of rare borderline thyroid tumors in pediatric patients is not well defined. Therefore, we aimed to present an illustrative assay of the pathological foundations underlying the sonographic appearance of benign, borderline, and malignant thyroid nodules in the pediatric population at a single tertiary thyroid center.

## 2 Material and methods

### 2.1 Patients

**Figure 1:**
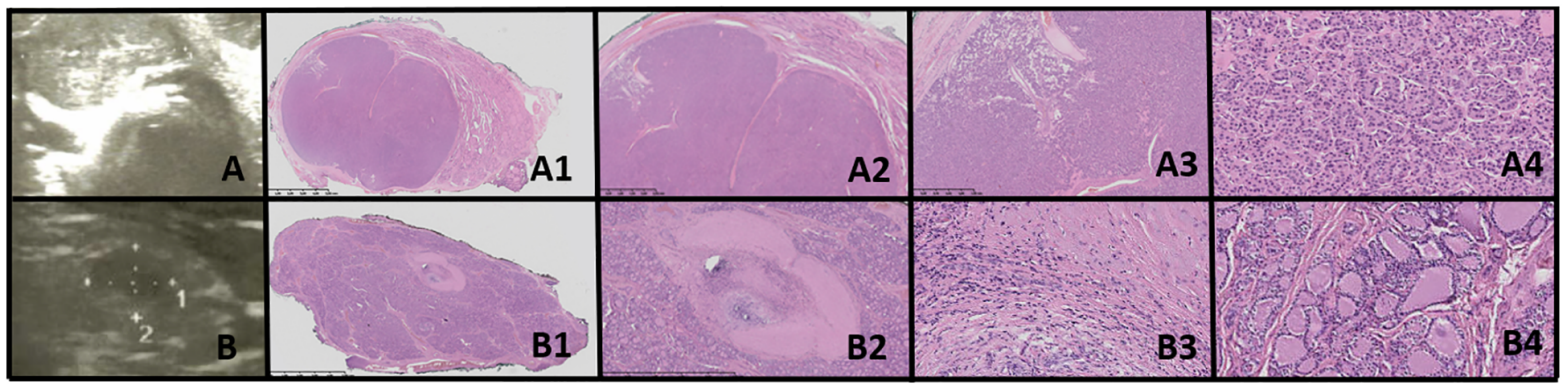
Dyshormonogenetic thyroid goiter (DHG). Columns represent: US and HE (magnification: A1, B1 ×5; A2, B2 ×50; A3, B3 ×1,000; and A4, B4 ×5,000). **(A)** Eighteen-year-old female patient; **(B)** 8-year-old female patient. US reveals a hypoechogenic nodule with well-defined borders and with hyperechogenic areas inside the nodule. In HE fibrosis, hemorrhages and inflammatory granulation tissue are seen. The structure is microfollicular and the nuclei are slightly enlarged and rarely overlap (A4, B3).

**Figure 2:**
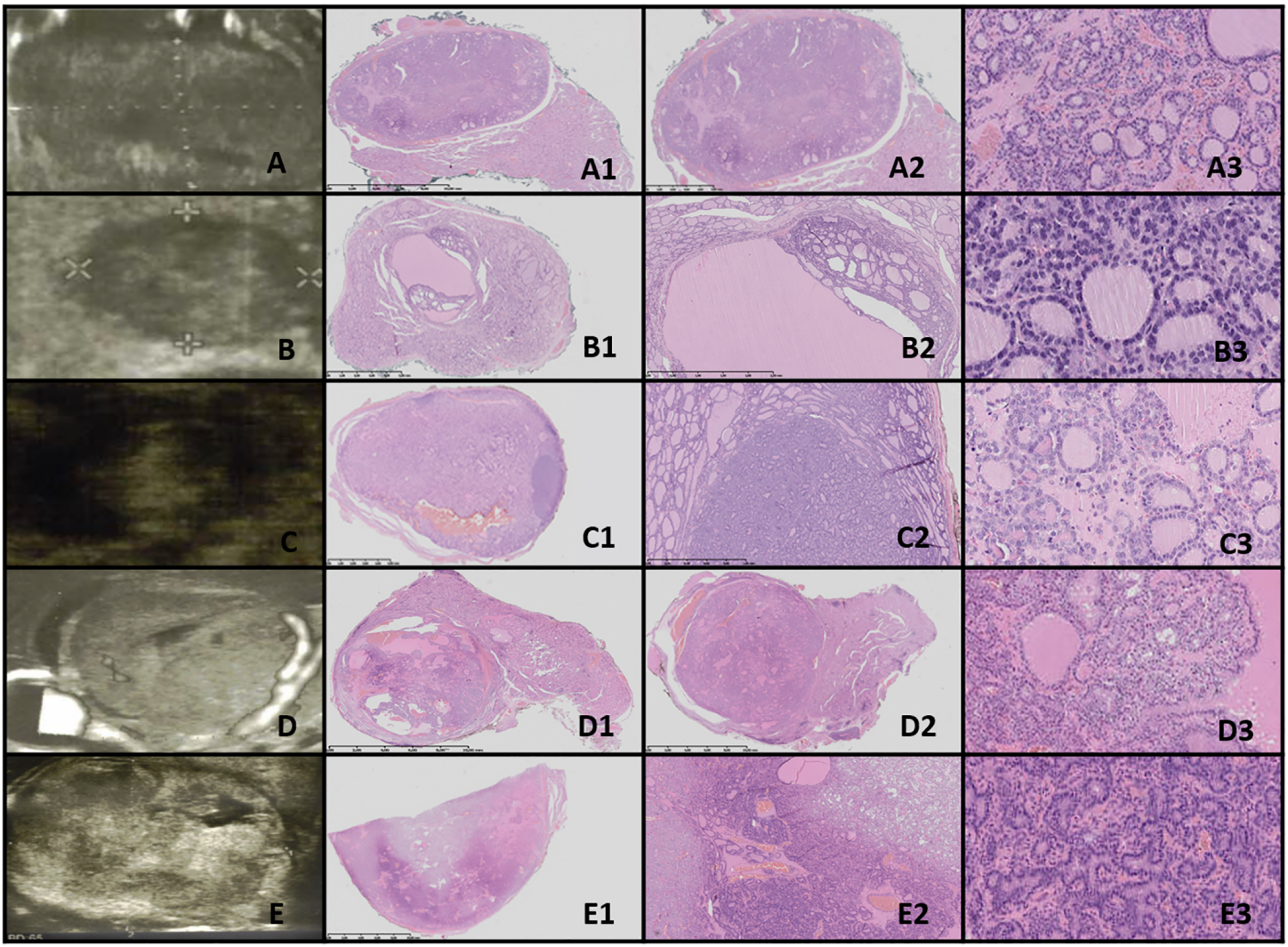
Thyroid follicular nodular disease (TFND). Columns represent US and HE (magnification A1–E1 ×5; A2–E2 ×50; and A3–E3 ×5,000). **(A)** Sixteen-year-old male patient with euthyroid goiter; **(B)** 14-year-old male patient with euthyroid goiter; **(C)** 18-year-old female patient with euthyroid goiter; **(D)** 18-year-old female patient with euthyroid goiter; **(E)** 15-year-old female patient with euthyroid goiter. In US, TFND is usually seen as a well-defined hyperechogenic nodule, surrounded by a hypoechogenic “halo” rim with mixed hypervascularity. In HE, macrofollicular (large follicles filled with pink colloid), medium-sized, and microfollicular structures are seen. Focally small fibrosis, hemorrhages, and papillary-like features are seen. The nuclei are a mixture of normotypical, slightly enlarged, and elongated, and they rarely have grooves. In patient **(E)**, ischemia (shrunk cells partially detached from the tissue matrix) in the central area of the nodule and clear-cell change are seen.

**Figure 3:**
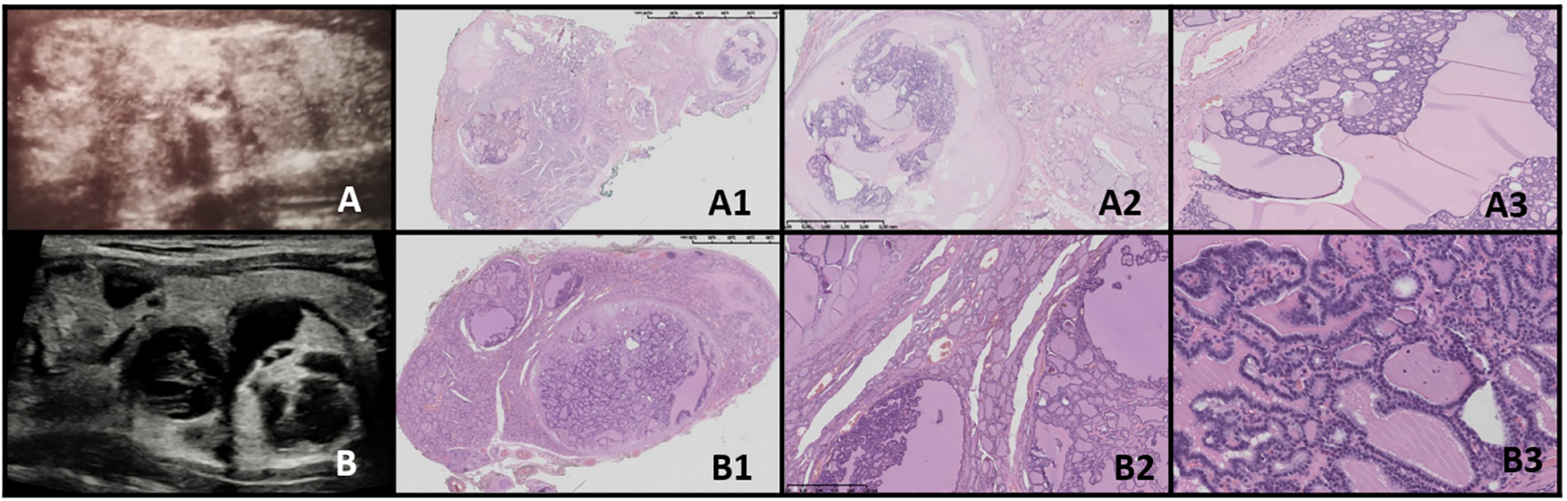
Thyroid follicular nodular disease (TFND) in patients with DICER1 syndrome. Columns represent US and HE (magnification: A1, B1 ×5; A2, B2 ×50; and A3, B3 ×5,000). **(A)** Seventeen-year-old female patient with euthyroid TFND; **(B)** 12-year-old male patient with euthyroid TFND. US shows multinodular goiter composed of hyper/isoechogenic solid-cystic nodules with macrocalcifications, especially in patient **(A)** In HE, the whole thyroid is built up by many hypocellular nodules filled with pink colloid. The hyperplastic nodules present a small, medium, and large vesicular structure and focally papillary arrangement (intrafollicular centripetal growth). Some of the nodules show areas of non-specific granulation, fibrosis, single calcifications, and a mixed-cellular inflammatory infiltrate with foamy macrophages containing hemosiderin. The remaining thyroid parenchyma is slightly congested.

**Figure 4:**
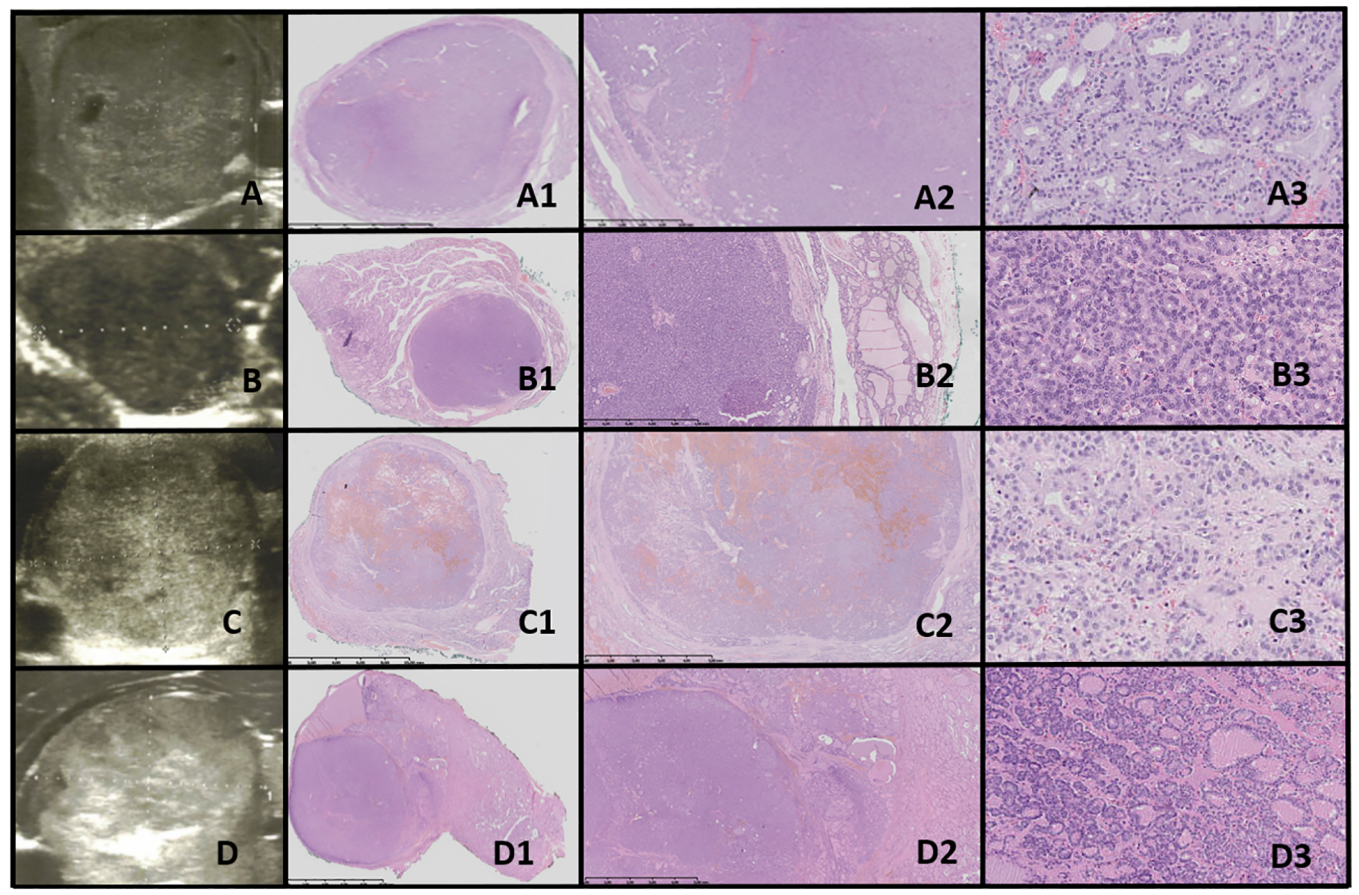
Thyroid follicular adenoma (TFA). Columns represent US and HE (magnification: A1–D1 ×5, A2–D2 ×50, and A3–D3 ×5,000). **(A)** Sixteen-year-old female patient with nodule found on US; **(B)** 16-year-old male patient with a nodule found on US; **(C)** 16-year-old female patient with euthyroid goiter with hoarseness; **(D)** 18-year-old male patient with euthyroid goiter with hoarseness. On US, a solitary, solid, round to oval, hypo/hyper/isoechogenic nodule is seen with well-defined hypoechogenic “halo” borders. On HE, the nodule is encapsulated, and the capsule is focally thickened and irregular. Pathological examination reveals no invasion through the capsule, the follicles inside a nodule are tightly packed, and the thyroid follicles adjacent to the nodule are constricted, larger (containing more colloid), but elongated. The nuclei are enlarged, with clearing and often overlap.

**Figure 5:**
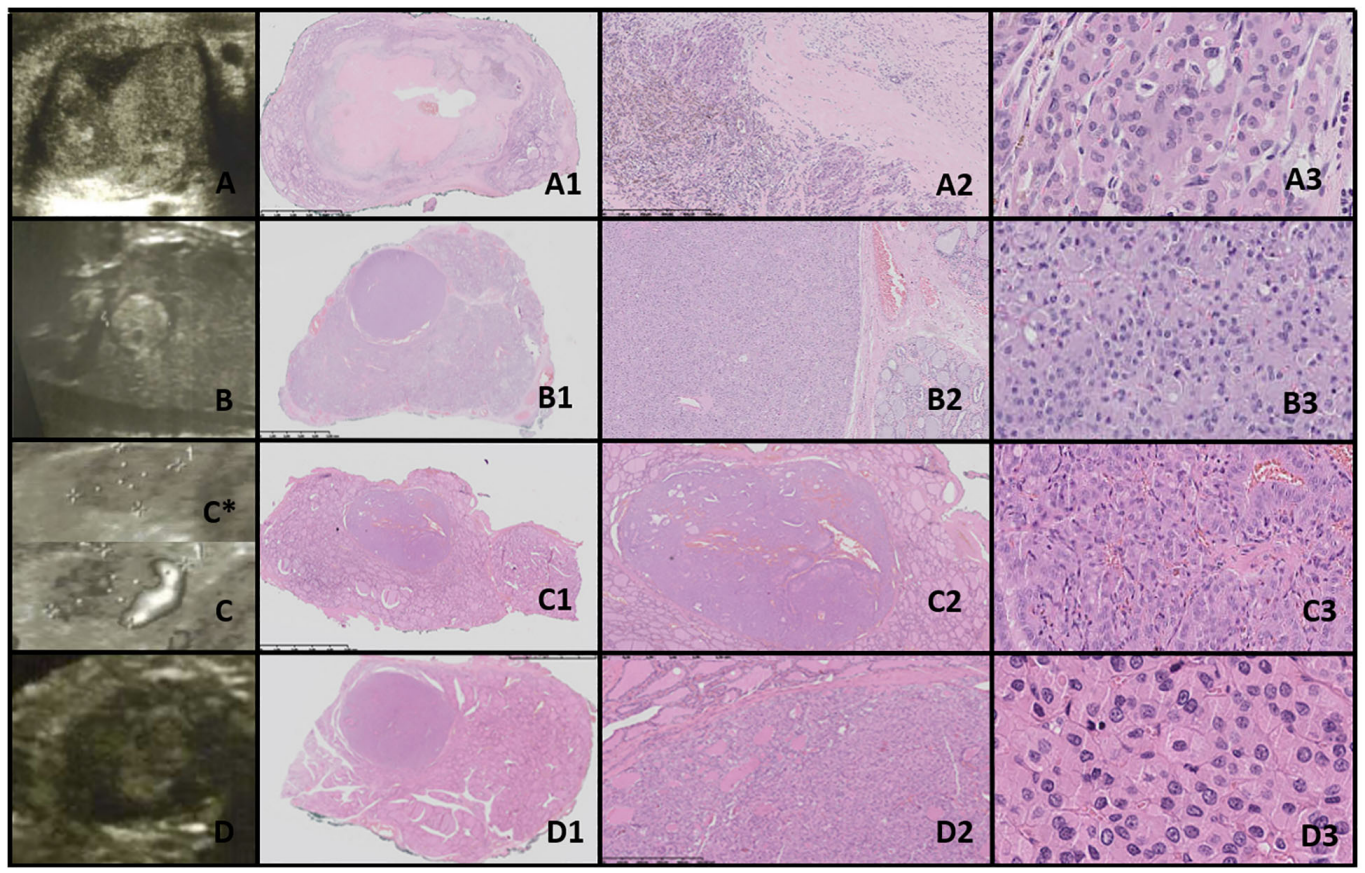
Oncocytic cell adenoma (OCA; Hürthle cell adenoma). Columns represent US and HE (magnification: A1–D1 ×5, A2–D2 ×50, and A3–D3 ×5,000). **(A)** Seventeen-year-old male patient with goiter; **(B)** 16-year-old female patient with a nodule found on US; **(C)** 17-year-old male patient with a nodule found on US; **(D)** 16-year-old male patient with a nodule found on US. US (**C, D** with power doppler, C*-without power doppler) reveals hyperechogenic nodules with small foci of hypoechogenic areas and increased mixed-type vascularity. HE examination reveals densely packed eosinophilic cells, and the hypoechogenic foci represent granular inflammatory tissue. Some of the cases might present with advanced fibrosis or contain medium-sized vessels (HE) and hyperperfusion in US (**A, C**, particularly). Cells are pleomorphic, with enlarged nuclei with prominent nucleoli.

**Figure 6:**
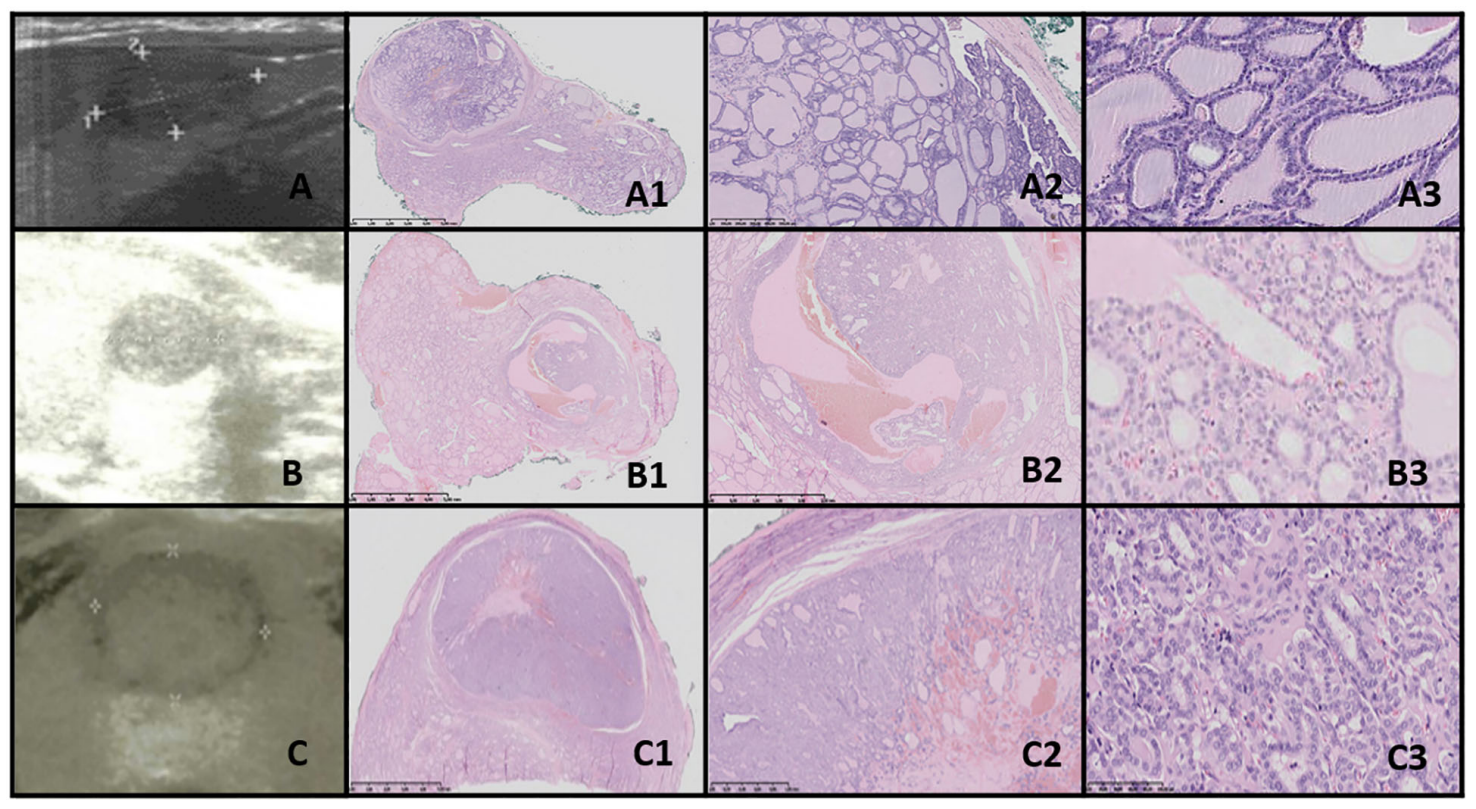
NIFTP. Columns represent US and HE (magnification: A1–C1 ×5, A2–C2 ×50, and A3–C3 ×5,000). **(A)** Fifteen-year-old male patient with a nodule found on US; **(B)** 12-year-old male patient with a nodule found on US; **(C)** 14-year-old male patient with a nodule found on US. US reveals a small, well-defined hypoechogenic nodule with acoustic (posterior) enhancement. HE reveals follicular structures, from micro- to macrofollicles; the nodule is round or oval, well-defined with or without fibrotic capsule, and there is an absence of capsular and vascular invasion. The nuclei have a set of PTC features (focally grade 3).

**Figure 7:**
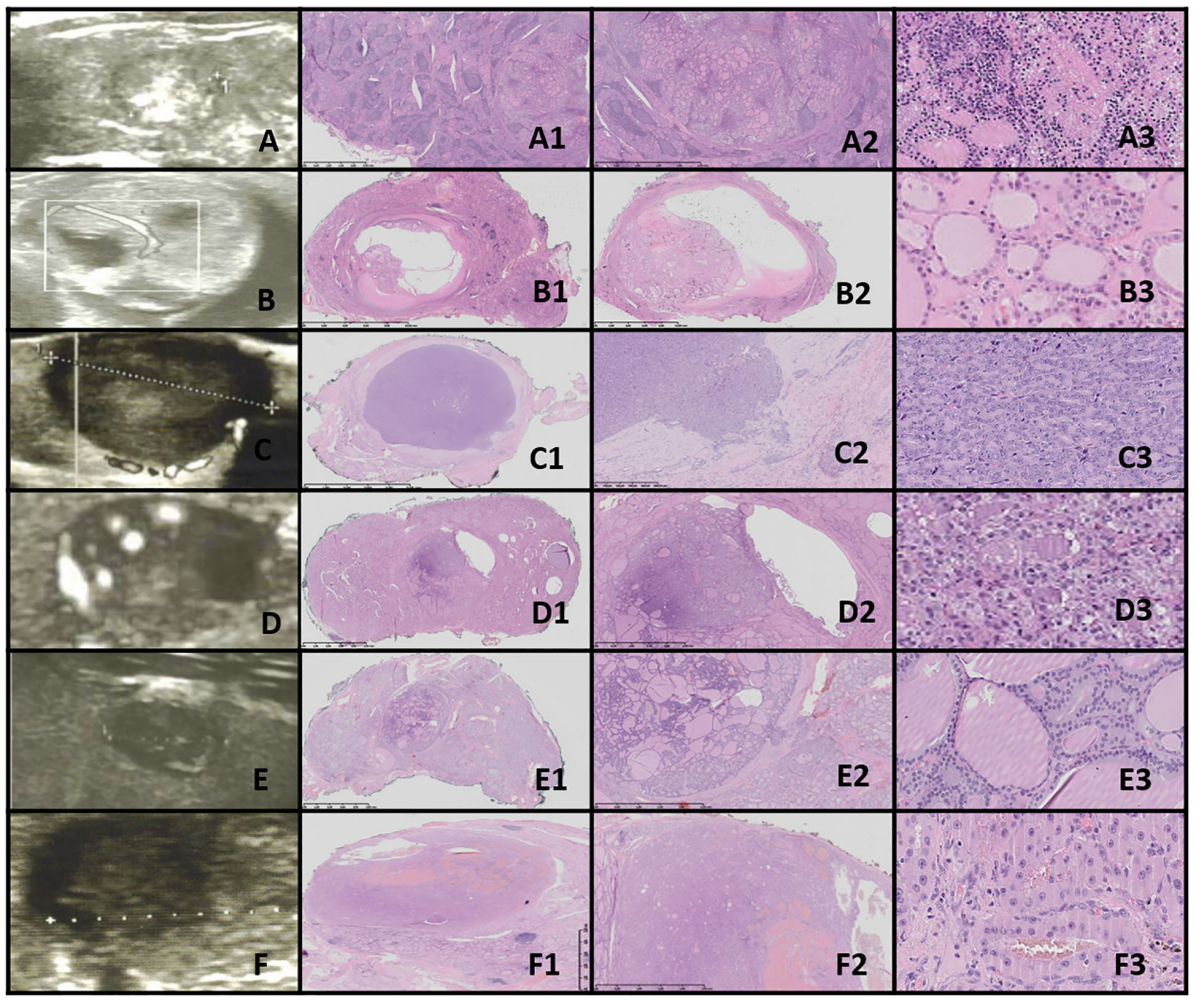
FT-UMP. Columns represent US and HE (magnification: A1–F1 ×5, A2–F2 ×50, and A3–F3 ×5,000). **(A)** Seventeen-year-old female patient; **(B)** 17-year-old female patient; **(C)** 18-year-old female patient; **(D)** 16-year-old female patient; **(E)** 16-year-old female patient; **(F)** 16-year-old male patient. In all patients, a nodule was found on US. US revealed small, foremost well-defined nodules with hypo- and hyperechogenic areas; however, focally the nodules’ borders are hard to define. Vascularization is mixed in the nodules. HE reveals follicular structures, from micro- to macrofollicles, and the nodule is round or oval with uncertain foci of capsule invasion. There is absence of PTC nuclear features.

**Figure 8:**
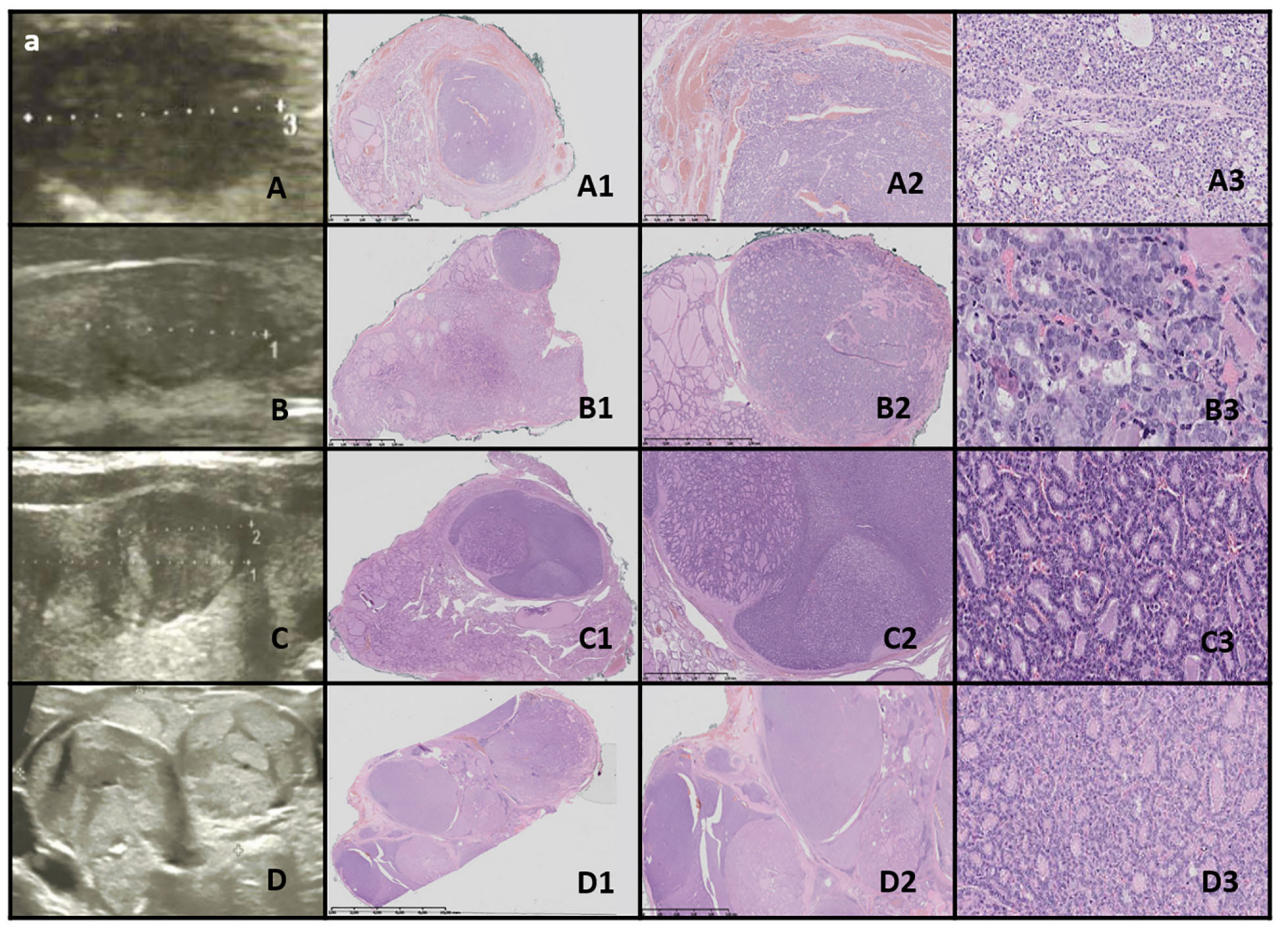
WDT-UMP. Columns represent US and HE (magnification: A1–D1 ×5, A2–D2 ×50, and A3–D3 ×5,000). **(A)** Seventeen-year-old male patient; **(B)** 16-year-old male patient; **(C)** 15-year-old female patient; **(D)** 17-year-old female patient. US reveals a medium-sized, quite well-defined hypoechogenic nodule in some cases with additional hyperechogenic areas. HE reveals follicular structures, predominantly microfollicles (hypoechogenic), and the nodule is oval, foremost well-defined but with the presence of capsular and vascular invasion, and the nuclei have PTC features.

**Figure 9:**
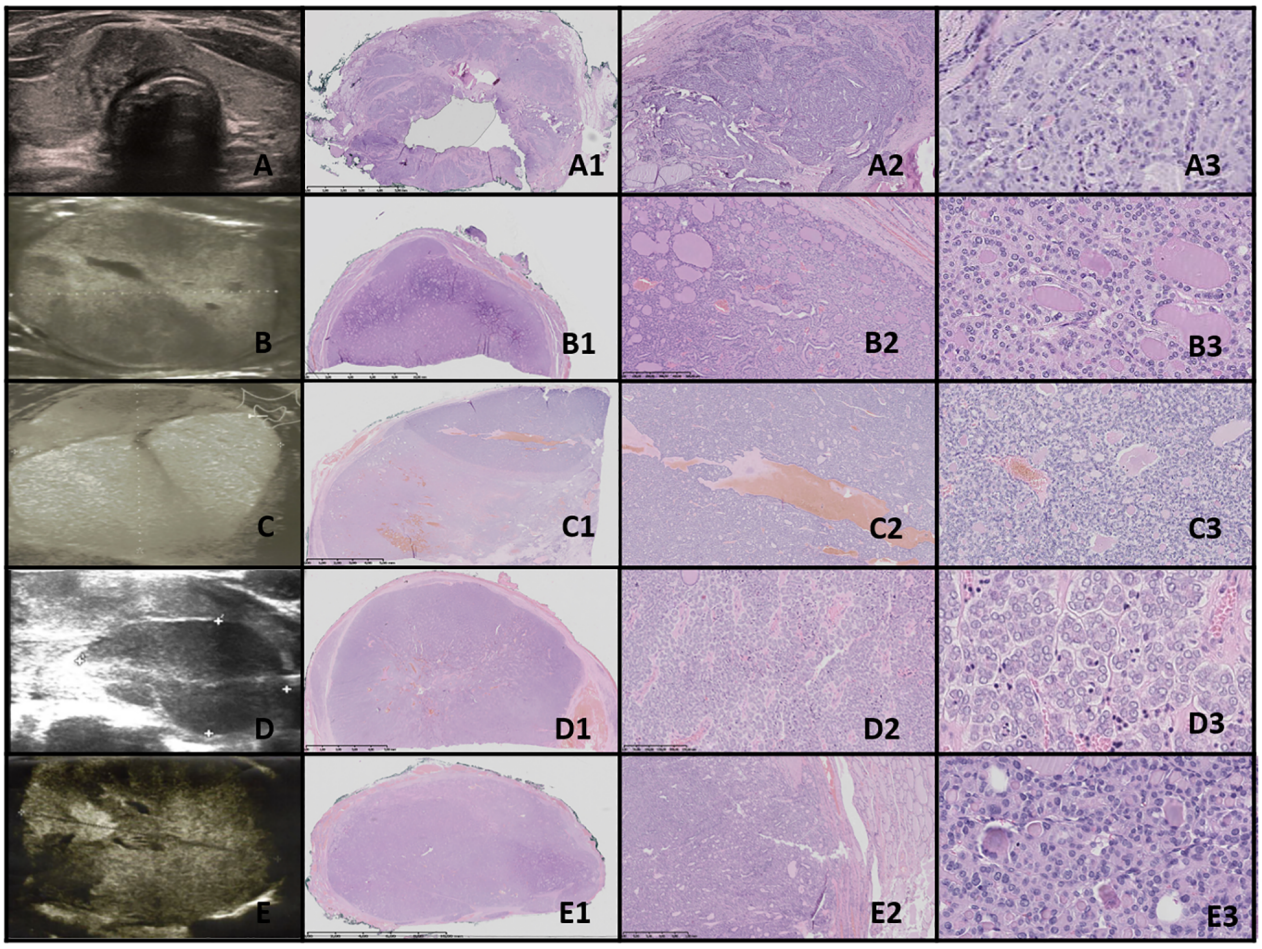
DTC and PDTC. Columns represent US and HE (magnification: A1–E1 ×5, A2–E2 ×50, and A3-E3 ×5,000). **(A)** Fifteen-year-old female patient with PTC; **(B)** 11-year-old female patient with FTC; **(C)** 16-year-old male patient with FTC; **(D)** 17-year-old female patient with PDTC; **(E)** 16-year-old female patient with PDTC. PTC **(A)**. US shows an irregular contoured, hypoechogenic nodule. In HE, the mixture of different-sized follicles built up from polymorphic cells with nuclei of “glassy” clearing and with grooves, invading through the capsule. FTC **(B, C)** and PDTC **(D, E)**. US shows large, hypo- and hyperechogenic nodules; however, although the outlines might seem to be well-defined, there are quite large areas of uncertain borders consisting of small hyperechogenic fragments. HE reveals follicular structures, from micro- to macrofollicles, which invade through the capsule and/or there is an angioinvasion **(D)**. The cells are pleomorphic and have large, overlapping nuclei. In PDTC, a set of neuroendocrine differentiation (salt-and-pepper nuclei) and trabecular structures can be found.

**Figure 10:**
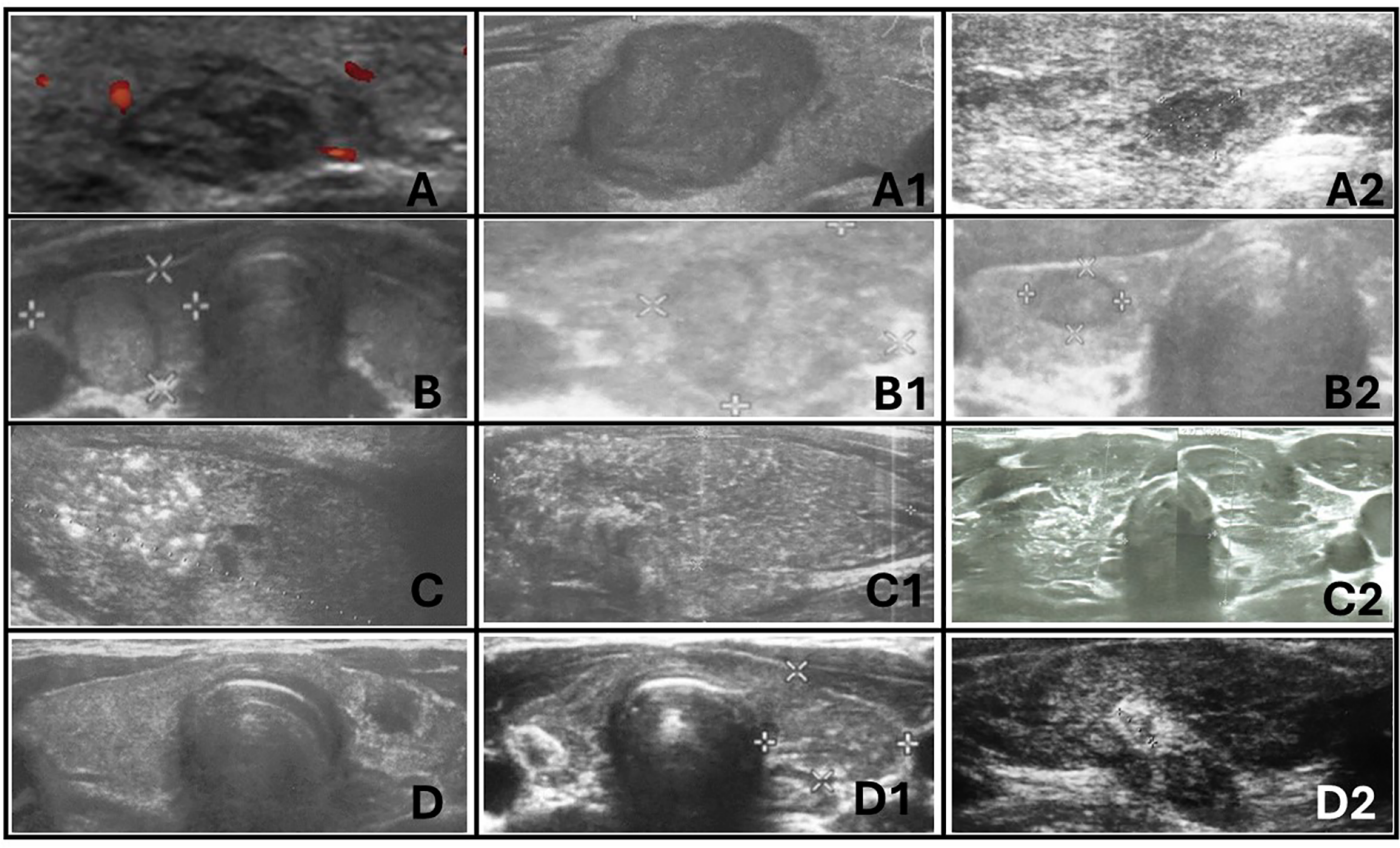
Ultrasonographic spectrum of papillary thyroid carcinoma in pediatric patients. **(A-A2)** hypoechogenic lesions with irregular margins; no increased vascularization in **(A)** and **(A2)**; no microcalcifications in **(A-A2)**; the shape is irregular oval, composition is solid. All patients with autoimmune thyroiditis (AIT). **(B-B2)** iso- and hypoechogenic lesions with `halo`; increased mixed vascularization and no microcalcifications in **(B-B2)**; the shape is taller than wider or wider than taller, composition is solid. Only **(B1)** with AIT. **(C-C2)** represents diffuse sclerosing variant of PTC. Extrathyroidal invasion is seen on **(C1)**. Vascularisation was increased in all lesions. All patients with AIT. **(D-D2)** represents hypoechogenic lesions surrounded by hyperechogenic irregular margin (histopathologically reported as fibrosis) in patients with autoimmune thyroiditis. No microcalcifications were seen but vascularization was increased in all lesions.

For this illustrative study, we selected 35 well-documented cases that provided comprehensive data, including hormonal profiles, clinical information, and high-quality US images, which we could optimally match with high-resolution histopathological scans (**Table 1**; **Figures 1**–**9**). We also chose representative, high-quality US images from 12 patients, depicting all types of papillary carcinoma encountered in our thyroid center (**Table 1**; **Figure 10**). Our selection criteria were solely based on the best-documented, educational, and representative cases from our image collection.

**Table 1:** Clinical evaluation of presented pediatric patients with thyroid nodules.

Patient	Sex	Age (years)	Clinical features of thyroid and/or risk group	TSH μIU/mL *N*: 0.3–4.0	fT4 pmol/L *N*: 10–25	TPOAb IU/ml *N* < 30	TgAb U/ml N<20	FNAB Bethesda score	Extent of surgery	Pathology
Figure 1										
A	F	18	Congenital hypothyreosis (CH) with goiter (two nodules in left lobe)	1.3	17.4	<30	<20	III/II (first FNAB) III/III (repeated FNAB)	L	Dyshormonogenetic goiter
B	F	8	Congenital hypothyreosis (CH) with goiter	1.7	16.8	<30	<20	III	L	Dyshormonogenetic goiter
Figure 2										
A	M	16	Goiter	3.5	0.9	1.5	0.1	III	L	TFND
B	M	14	Goiter, diabetes	1.5	15.6	<30	<20	IV	L	TFND
C	F	18	Goiter	1.6	15.6	<30	<20	III	L+I	TFND
D	F	18	Goiter	1.2	15.8	<30	588	IV	L	TFND
E	F	15	Goiter with a 5-cm nodule	1.7	16.3	<30	<20	III	L	TFND
Figure 3										
A	F	17	Goiter 68 mL	1.9	16	<30	<20	III	TT	TFND DICER+
B	M	12	Goiter 32 mL	1.2	17	<30	<20	III	TT	TFND DICER+
Figure 4										
A	F	16	Ultrasound evaluation	1.7	13	<0.8	<6.4	IV	L	Thyroid follicular adenoma
B	M	16	Ultrasound evaluation	4.2	12.9	<30	<20	III	L	Thyroid follicular adenoma
C	F	16	Goiter with hoarseness	1.3	14.2	<30	<20	II	L+I	Thyroid follicular adenoma
D	M	17 11/12	Goiter with hoarseness	1.2	15.4	<30	<20	IV	L+I	Thyroid follicular adenoma
Figure 5										
A	M	17	Goiter with a 20×17×23 mm nodule	2.12	15.3	<30	<20	III	L	Oncocytic adenoma
B	F	16	Brain radiotherapy (ALL), ultrasound surveillance	2.0	13.5	<30	<20	II, III, IV	TT	Oncocytic adenoma
C	M	17	Ultrasound evaluation	1.0	14.8	<30	<20	V	L	Oncocytic adenoma
D	M	16	Ultrasound evaluation	1.2	15.2	<30	<20	III	L	Oncocytic adenoma
Figure 6										
A	M	15	Ultrasound evaluation	5.2	16.6	<30	<20	III	L	NIFTP
B	M	12	Ultrasound evaluation	2.8	14.4	<30	<20	III	L	NIFTP
C	M	14	CH, dyshormonogenetic MNG	1.5	17.7	<30	<20	III	TT	NIFTP
Figure 7										
A	F	17.2	Ultrasound evaluation	1.5	16.6	34.2	20	III	TT	FT-UMP
B	F	17	AIT, ultrasound surveillance	1.5	11.6	30	1,217.8	III	L	FT-UMP
C	F	18	AIT, ultrasound surveillance	1.9	16.8	58	1,500	III	L	FT-UMP
D	F	16	Ultrasound evaluation	1.3	15.5	<30	<20	III	L	FT-UMP
E	F	16	Brain radiotherapy (ALL), ultrasound surveillance	2.0	13.5	<30	<20	II, III, IV	TT	FT-UMP
F	M	16	Ultrasound evaluation	1.2	15.2	<30	<20	III	L	FT-UMP
Figure 8										
A	M	17	ALL, ultrasound surveillance	4.3	14.8	<30	<20	III	TT	WDT-UMP
B	M	16	2014 RTx total body, BMT (CGD), ultrasound surveillance	2.2	14.5	<30	<20	III	L+I	WDT-UMP
C	F	15	Goiter	2.3	1.04	1.7	0.1	I, II, III	L	WDT-UMP
D	F	17	Goiter with hoarseness	0.6	14.7	>600	>100	II	L	WDT-UMP
Figure 9										
A	F	15	Goiter	1.5	16.6	<30	<20	VI	TT	PTC
B	F	11	Goiter with hoarseness	1.5	14.5	<30	<20	III	TT	FTC
C	M	16	Goiter, 6 cm nodule	2.2	18	<30	<20	III	TT	Encapsulated FTC with angioinvasion
D	F	17	Goiter	1.46	15.3	<30	<20	VI	TT	PDTC
E	F	16	Goiter	1.4	13.8	<30	<20	VI	TT	PDTC
Figure 10										
A	F	17	US screening in thyroid clinic	1.4	15.7	>2,000	>1,000	VI	TT	PTC
A1	F	17	US screening in thyroid clinic	2.1	16.5	547.1	124.1	VI	TT	PTC
A2	F	10	US screening in thyroid clinic	1.2	12.9	143.4	93.4	VI	TT	PTC
B	M	10	US screening in thyroid clinic	2.6	14.5	<30	<20	VI	TT	PTC
B1	F	17	US screening in thyroid clinic	5.01	11.4	1,272.1	–	VI	TT	PTC
B2	M	16	US screening in thyroid clinic	2.3	14.7	<30	<20	VI	TT	PTC
C	F	12	US screening in thyroid clinic	<0.02	fT3 > 30.8 [*n*: 3.6–8.6] pmol/L fT4 70.1	<30	TgAb < 20 TRAb 4.0 IU/L [*n* < 1]	VI	TT	PTC
C1	F	14	US screening in thyroid clinic	0.02	fT3 13.4 [*n:* 3.6–8.6] pmol/L fT4 31.6	<30	TgAb < 20 TRAb 5.2 IU/L [*n* < 1]	VI	TT	PTC
C2	F	13	US screening in thyroid clinic	1.5	15.9	>1,000	>6,000	VI	TT	PTC
D	F	14	US screening in thyroid clinic	2.9	16.7	925.9	21.1	VI	TT	PTC
D1	F	8	US screening in thyroid clinic	0.9	17.1	748.2	22	VI	TT	PTC
D2	F	12	US screening in thyroid clinic	1.5	16.5	290.8	88.5	VI	TT	PTC

ALL, acute lymphocytic leukemia; CGD, chronic granulomatous disease; BMT, bone marrow transplantation; RTx, radiotherapy; AIT, autoimmune thyroiditis; CH, congenital hypothyreosis; MNG, multinodular goiter; L, lobectomy; L+I, lobectomy with isthmectomy; TT, total thyroidectomy; TFND, thyroid follicular nodular disease; PTC, papillary thyroid carcinoma; FTC, follicular thyroid carcinoma; PDTC, poorly differentiated thyroid carcinoma; WDT-UMP, well-differentiated tumor of unknown malignant potential; FT-UMP, follicular tumor of unknown malignant potential; NIFTP, noninvasive follicular thyroid neoplasm with papillary-like nuclear features.

This cohort of 47 patients was selected from the group of 262 pediatric patients (196 female patients; mean age of 13.1 years; age range, 6 to 18 years) who were referred for thyroid surgery to the University Children’s Hospital in Krakow, a major tertiary pediatric center in Southeastern Poland, between 2010 and 2023.

### 2.2 Methods

The retrospective analysis of medical records involved evaluating thyroid function, as well as ultrasound and histopathological characteristics in patients with thyroid nodules. All hormonal and immune assessments were routinely conducted at the Department of Biochemistry, University Children’s Hospital in Krakow, Poland. These assessments were performed on a single fasting blood sample, as previously described (18, 19). Thyroid-stimulating hormone (TSH) and free thyroxine (fT4) levels were measured using immunoassay methods with an ADVIA Centaur analyzer, while thyroid peroxidase antibodies (TPOAb) and thyroglobulin antibodies (TgAb) were assessed via radioimmunoassay using a Brams machine. All assessments were completed prior to the initiation of therapy, including levothyroxine or antithyroid drug treatment when required (except in patients with congenital hypothyroidism) and before any surgical intervention. Molecular analyses were routinely performed in cases of suspected genetic syndromes.

Thyroid ultrasonography (US) was conducted by certified pediatric endocrinologist and surgeon with significant experience in pediatric US (DJ > 20 years and AKW > 15 years). The examinations were performed using high-resolution systems: Voluson 730 GE Medical System (8–12 MHz linear-array transducer), Philips Epiq5 (L12-5 linear transducer), Philips iE22 (L11-3 linear transducer), and Samsung HS40 (LA3-16AD transducer), as previously described (18, 19). The analysis included ultrasound features of the thyroid gland based on the EU- TIRADS PL 2022 classification (Polish update of EU-TIRADS 2017) (20, 21) (**Table 2**).

**Table 2:** EU-TIRADS-PL classification (20).

EU-TIRADS-PL category	Ultrasonographic features	Risk of malignancy	Indications for biopsy and/or further ultrasound monitoring
1	No nodules	Close to 0%	Ultrasound follow-up depending on clinical risk factors
2	Pure cysts Spongiform nodules	Close to 0%	FNAB not recommended (exception: therapeutic biopsy in symptomatic patients, e.g., cyst drainage); ultrasound follow-up depending on clinical risk factors
3	Normal/isoechoic or hyperechoic Ovoid or round shape Smooth margins No features of category 5	2%–4%	FNAB ≥ 20 mm
4	Hypoechoic Ovoid or round shape Smooth margins No features of category 5	6%–17%	FNAB ≥ 15 mm
5	Presence of at least one of the following features: • Marked hypoechogenicity • Irregular shape • Non-parallel orientation • Irregular margins • Microcalcifications • Extrathyroidal invasion*	>26%*	FNAB ≥ 5 mm*

Modified based on 2017 EU-TIRADS guidelines (21). *Modifications in comparison with the EU-TIRADS classification are marked: in EU -TIRADS 5: >10 mm FNAB, <10 mm consider FNAB or active surveillance; in EU-TIRADS 5: high risk 26-87%; in EU-TIRADS-PL 5- additional features: irregular shape and extrathyroidal invasion. Legend: FNAB — fine-needle aspiration biopsy.

Fine-needle aspiration biopsy (FNAB) results were categorized according to the 2023 (an update of 2017) Bethesda System for Reporting Thyroid Cytopathology (TBSRTC) (22, 23). Surgical procedures included lobectomy, lobectomy with isthmectomy, or total thyroidectomy with central and, when necessary, lateral lymph node dissection (**Table 1**).

Histopathological evaluations were performed at the Department of Pathology, University Children’s Hospital, and the Department of Pathomorphology, Jagiellonian University in Krakow, with MK serving as the responsible pathologist. Hematoxylin and eosin (HE)-stained tissue sections (deparaffinized, cut at 3.5 µm thickness) were scanned using the NanoZoomer SQ Hamamatsu at 400× magnification after routine diagnosis of thyroid nodules. Images were captured from the scans, with a scale bar positioned in the lower left corner.

Preoperative US images were analyzed with histopathology findings, with a focus on the nodule’s shape, composition, echogenicity, margin characteristics, vascularity, extrathyroidal invasion, and presence of calcifications.

This study was approved by the relevant institutional review board (The Ethics Committee of the Jagiellonian University opinion number:118.0043.1.103.2024 issued on 19 April 2024). Written informed consent was obtained from all participants and/or their parents. Written informed consent was obtained from the individual(s) and minor(s) legal guardian/next of kin for the publication of any potentially identifiable images or data included in this article.

## 3 Results

### 3.1 General overview

Between 2010 and 2023, 262 pediatric patients were referred for thyroid surgery at a major tertiary pediatric center. Histopathological diagnoses were updated according to the 2022 WHO Classification of Thyroid Tumors, revealing that 140 patients (53.4%) had benign nodules, 18 patients (6.9%) had borderline nodules, and 104 patients (39.7%) had malignant nodules (2). Among the 262 patients, the diagnoses were as follows: TFND in 84 (32.1%), thyroid follicular adenoma (TFA) in 25 (9.5%), OCA in 4 (1.5%), large thyroid cysts in 4 (1.5%), dyshormonogenetic goiter (DHG) in 2 (0.8%), therapy-resistant Graves’ disease in 21 (8.0%), NIFTP in 3 (1.1%), WDT-UMP in 4 (1.5%), FT-UMP in 11 (4.2%), PTC in 89 (33.9%), invasive encapsulated follicular variant of PTC (IEFVPTC) in 1 (0.4%), FTC in 2 (0.8%), oncocytic carcinoma in 1 (0.4%), PDTC in 2 (0.8%), and medullary thyroid carcinoma (MTC) in 9 (3.4%).

A cancer predisposition syndrome was identified in four patients: one with Gardner syndrome and the columnar cell subtype of PTC, two with DICER1 syndrome and TFND, and one with Cowden syndrome and oncocytic carcinoma. For this illustrative review, we selected 35 representative cases that covered the full spectrum of gray-scale US features observed in our clinic, correlating these with histopathological findings (see **Figures 1**–**9**; **Tables 1**, **3**–**5**). Additionally, we included high-quality, illustrative US images from 12 patients that demonstrate the diverse sonographic presentations of PTC encountered at our center (**Figure 10**; **Tables 1**, **3**).

**Table 3:** Ultrasonographic features of benign, borderline, and malignant lesions in the presented group of pediatric patients.

Ultrasound feature of the nodule		Benign					Borderline			Malignant
		DHG	TFND DICER−	TFND DICER+	TFA	OA	NIFTP	FT-UMP	WDT-UMP	DTC PDTC
Composition	Solid-cystic	−	+/−	++	−	−	+/−	+/−	−	−
	Solid	+	+	−	+	+	+	+	+	+
Echogenicity	Anechoic	−	−	+	−	−	−	−	−	−
	Hyper/isoechoic	−	+	+	+	+	−	+	−	+
	Mixed hypo/hyperechoic	+	+	+	+	+	+	+	+	+
	Hypoechoic	−	−	−	+	−	+	+	+	++
Orientation of the nodule	Parallel (wider than taller)	+	+	+	−	+	+	+	+	+
	Non-parallel (taller than wider)	−	−	++	+	+	+/−	−	+	++
Margin	Smooth	+	+	+	+	+	+	+	+	+
	Lobulated	−	−	−	−	−	−	−	−	+
	Irregular	−	−	−	−	−	−	−	−	+
	Ill-defined	+	−	−	−	−	−	−	−	+
Calcification	Macrocalcification > 1 mm	−	−	+	−	−	−	−	−	−
	Microcalcification <1 mm	−	−	−	−	−	−	−	−	+
Vascularization	Peripheral	+	+	+	+	−	+	−	−	+
	Mixed	−	+	+	+	+	+	+	+	+
	Central	−	−	+	+	−	+	+	+	+
Shaping of the gland and capsule	Present	+	+	+	+	+	+	−	+	+
Extrathyroidal invasion	Present	−	−	−	−	−	−	−	−	+
“Halo”	Present	+/−	+	+	+	+	+	+	+	+

DHG, dyshormonogenetic goiter; TFND, thyroid follicular nodular disease; TFA, thyroid follicular adenoma; OA, oncocytic adenoma; NIFTP, non-invasive follicular thyroid neoplasm with papillary-like nuclear features; FT-UMP, follicular tumor of uncertain malignant potential; WDT-UMP, well-differentiated tumor of uncertain malignant potential; DTC, differentiated thyroid carcinoma (PTC and FTC); PDTC-, poorly differentiated thyroid carcinoma.

**Table 4:** Histopathological features of benign, low-risk, and malignant lesions.

Histopathological features of the nodule		Benign					Borderline			Malignant
		DHG	TFND DICER−	TFND DICER+	TFA	OA	NIFTP	FT-UMP	WDT-UMP	DTC PDTC
Nuclei	Normotypical	++	++	−−	+	−	−−	−	−−	−−−
	Enlarged	+	+/−	+/−	+	+	+	+	+	+++
	Elongated	−	+	−	−	−	+	+	+	++
	Grooves	−	−	+/−	−/+	−/+	++	++	++	+++
	Clearing	−	−	+/−	+/−	+	++	++	++	+++
	Prominent nucleoli	−/+	−/+	+/−	−/+	++	−	−	−	−
Eosinophilic cytoplasm (intense pink)		+/−	−/+	+/−	+/−	+++	+/−	+/−	+/−	−/+
Well-defined and/or encapsulated nodule		+/−	+/−	+	+++	++	+++	++	++	−
Necrosis		−	−	−	−	−	−	−	−	+
Capsule invasion		−	−	−	−	−	−	+/−	+/−	+
Angioinvasion		−	−	−	−	−	−	+/−	+/−	++
Macrofollicular structure		++	++	++	+/−	+/−	+	+	+	−
Microfollicular structure		−/+	+/−	+	++	++	++	++	++	+
Papillary structure		−/+	−	−	−	−/+	−	−	−	++
Cellularity, nuclear crowding		−	−	+/−	+/−	+/−	+	+	++	+++
Atypia and polymorphism (of the benign and malignant, respectively)		−	−	+	−	−/+	+	+	+	++
Fibrosis		+/−	+/−	+	−	+	−	−/+	−/+	++
Hemorrhages/ischemia		+/−	+/−	+/−	+	++	−	−	−/+	−/+
Hyperplastic nodules		−	−	+	−	−	−	−	−	−
Intrafollicular centripetal growth		−	−	+	−	−	−	−	−	−
Macrocalcifications		+	+	+	++	++	−/+	−/+	−/+	+/−
Microcalcifications (Psammoma bodies)		−	−	−	−	−	−	−	−	+++
Congested parenchyma between the nodules		−	−	+	−	−	−	−	−	−

DHG, dyshormonogenetic goiter; TFND, thyroid follicular nodular disease; TFA, thyroid follicular adenoma; OA, oncocytic adenoma; NIFTP, non-invasive follicular thyroid neoplasm with papillary-like nuclear features; FT-UMP, follicular tumor of uncertain malignant potential; WDT-UMP, well-differentiated tumor of uncertain malignant potential; DTC, differentiated thyroid carcinoma (PTC&FTC); PDTC, poorly differentiated thyroid carcinoma.

**Table 5:** Pathologic basis of ultrasound features (shape, margin, echogenicity, “halo”, calcifications, and composition).

Feature	Sonography	Histopathology	Tumor type	Figures
Shape	Wider than taller, round	Horizontal growth	DHG TFND DICER− TFND DICER+ OA NIFTP FT-UMP WDT-UMP PTC	**Figure 1** **Figure 2** **Figure 3** **Figure 5** **Figure 6** **Figure 7** **Figure 8** **Figure 10B2**
	Taller than wider	Vertical growth	TFND DICER+ TFA WDT-UMP PTC	**Figure 3** **Figure 4** **Figure 8** **Figure 10**
Margin	Smooth	No invasion	DHG TFND DICER− TFND DICER+ TFA OA NIFTP FT-UMP WDT-UMP PTC	**Figure 1** **Figure 2** **Figure 3** **Figure 4** **Figure 5** **Figure 6** **Figure 7** **Figure 8** **Figure 10B2**
	Lobulated	Expansile growth, group of tumor cells “pushing” into surrounding follicles	PTC	**Figure 9A** **Figure 10A1**
	Irregular	Irregular	PTC	**Figure 10C-C2** **Figure 10D-D2**
Echogenicity	Marked hypoechoic	Tightly packed microfollicles or solid nests of tumor cells	PDTC PTC	**Figures 9D, E** **Figure 10A1, A2**
	Hypoechoic	Small-to-medium follicles	DHG TFND DICER− TFA OA NIFTP FT-UMP WDT-UMP PTC FTC	**Figure 1** **Figure 2** **Figures 4B, C** **Figure 5** **Figure 6** **Figure 7** **Figure 8** **Figure 9A** **Figures 9B, C**
	Isoechoic to hyperechoic	Normal to macrofollicles	TFND DICER− TFND DICER+ TFA OA FT-UMP WDT-UMP FTC PTC	**Figure 2** **Figure 3** **Figures 4A, D** **Figure 5** **Figure 7** **Figure 8** **Figures 9B, C** **Figure 10B**
Hypoechoic rim (halo)	Present	Capsule around normal-sized follicles or macrofollicles	DHG TFND DICER− TFND DICER+ TFA OA NIFTP FT-UMP WDT-UMP PTC	**Figure 1** **Figure 2** **Figure 3** **Figure 4** **Figure 5** **Figure 6** **Figure 7** **Figure 8** **Figure 10B**
	Absent	Tightly packed microfollicles	FT-UMP WDT-UMP	**Figure 7** **Figure 8**
Calcifications	Micro	Psammomatous small calcifications	PTC	**Figure 10C-C2**
	Macro	Coarse calcifications	TFND DICER+	**Figure 3A**
Composition	Isoechoic nodule with cystic areas	Macrofollicular nodule with cystic areas	TFND DICER+	**Figure 3**
	Solid	Solid, microfollicular, dense papillary or trabecular	DHG TFND DICER− TFA OA NIFTP FT-UMP WDT-UMP PTC, FTC, PDTC	**Figure 1** **Figure 2** **Figure 4** **Figure 5** **Figure 6** **Figure 7** **Figure 8** **Figures 9**, **10**

DHG, dyshormonogenetic goiter; TFND, thyroid follicular nodular disease; TFA, thyroid follicular adenoma; OA, oncocytic adenoma; NIFTP, non-invasive follicular thyroid neoplasm with papillary-like nuclear features; FT-UMP, follicular tumor of uncertain malignant potential; WDT-UMP, well-differentiated tumor of uncertain malignant potential; PDTC, poorly differentiated thyroid carcinoma.

Modified according to Yang et al. (1).

The clinical and endocrine evaluations of 47 patients are summarized in **Table 1**, which also details the risk factors associated with the development of thyroid nodules, such as prior radiotherapy and chemotherapy for primary cancers.

### 3.2 Hormonal assessment

All patients with benign, borderline, and malignant tumors were euthyroid prior to surgery, with or without levothyroxine or antithyroid therapy as needed. AIT was confirmed in 15 patients before surgery (10 with PTC, 3 with FT-UMP, 1 with TFND, and 1 with WDT-UMP) (**Table 1**).

### 3.3 Risk factors

Notable risk factors included congenital hypothyroidism with goiter in three patients, brain radiotherapy for acute lymphoblastic leukemia (ALL) in one patient with OCA, brain radiotherapy for ALL in one patient with FT-UMP, chemotherapy for ALL in one patient with WDT-UMP, and total body irradiation prior to bone marrow transplantation for chronic granulomatous disease in one patient with WDT-UMP. Additionally, nodular AIT was diagnosed in 10 patients with PTC, 3 patients with FT-UMP, 1 patient with TFND, and 1 patient with WDT-UMP.

### 3.4 Ultrasound features

The ultrasonographic features of the subgroups are presented in **Table 3** and in **Figures 1**–**10**. The evaluation included nodule composition, echogenicity, orientation, margin, calcifications, vascularization, gland and capsule shape, extrathyroidal invasion, and the presence of a “halo”. No distinct ultrasonographic patterns were identified to clearly differentiate benign, borderline, and malignant lesions. However, certain features were observed exclusively in malignant nodules, including microcalcifications, marked hypoechogenicity, lobulated or irregular ill-defined margins, and extrathyroidal invasion (**Figures 9**, **10**; **Table 3**). **Figure 10** displays various US images of pediatric PTC, highlighting different presentations: hypoechogenic nodules with irregular or lobulated margins (A–A2), iso/hyperechogenic nodules with a halo (B–B2), nodules that are wider than taller (B1 and B2), diffuse sclerosing subtype (C–C2), and hypoechoic nodules with a surrounding hyperechogenic margin (D–D2). Apart from the C–C2 lesions, no microcalcifications were observed on US. As shown in image A, no increased vascularization was observed in small lesions.

### 3.5 Fine-needle aspiration biopsy

FNAB was performed in all nodules (**Figures 1**–**10**). The results of FNAB are presented in **Table 1**.

### 3.6 Surgical outcome

Total thyroidectomy was the initial approach for patients with a high suspicion of malignancy (**Table 1**; **Figures 9**, **10**). Lobectomy, with or without isthmectomy, was more commonly selected as the first option for Bethesda category III cases and, less frequently, for category IV cases (**Table 1**). Surgical decisions were informed by FNAB results, as well as a comprehensive dataset that included patient history, age, gender, symptoms (e.g., large goiter, hoarseness), risk factors, US findings, and tumor growth potential, as previously described by Januś et al. (**Table 1**) (24).

### 3.7 Histopathological assessment

Histopathology remains the gold standard for differentiating and diagnosing thyroid lesions.

**Table 4** and **Figures 1**–**9** present the histopathological features of benign, borderline, and malignant lesions. The assessment included nuclear characteristics, cytoplasmic features, nodule margins, presence of necrosis, capsular and vascular invasion, tissue structure, cellularity, presence of calcifications, and characteristics of the surrounding thyroid parenchyma.

### 3.8 Ultrasound–histopathological general considerations

**Table 5** presents the pathological basis of the US features observed in the study patients, including shape, margin, echogenicity, “halo” appearance, calcifications, and composition.

Thyroid nodule sections stained with HE were compared with corresponding thyroid US images. The colloid stained pink with eosin, while the nuclei of the follicular cells stained blue with hematoxylin, as previously described (1). Follicle size was inversely related to echogenicity: microfollicular nodules, with high nuclear density, appeared blue on HE sections and markedly hypoechoic on gray-scale US, whereas macrofollicular nodules, with low nuclear density, appeared pink on HE sections and were isoechoic or hyperechoic on US (1). The fibrous tissue capsule attenuated sound waves, presenting as a hypoechoic rim on US, particularly when the nodule had higher echogenicity (1). The margin characteristics of thyroid nodules observed in HE sections corresponded closely with the US findings, as reviewed by Yang et al. (1).

### 3.9 Ultrasound–histopathological evaluation within the subgroups

#### 3.9.1 Benign thyroid nodules

##### 3.9.1.1 Dyshormonogenetic goiter

In both of our patients with DHG, US imaging revealed an enlarged thyroid with solid hypoechoic nodules. Pathological examination in both cases revealed fibrosis, hemorrhage, and inflammatory granulation tissue. The thyroid architecture was microfollicular, with slightly enlarged and infrequently overlapping nuclei, confirming the diagnosis of DHG (**Figure 1**).

##### 3.9.1.2 Thyroid follicular nodular disease (multinodular goiter)

Ultrasonographic evaluation revealed that TFND nodules were oval, isoechoic to hyperechoic, with a surrounding hypoechoic halo. Histological evaluation demonstrated variably sized dilated follicles with flattened to hyperplastic epithelium, with non-nodular thyroid tissue appearing reduced and compressed (**Figure 2**).

##### 3.9.1.3 TFND in DICER1 syndrome

The US revealed multinodular goiter (MNG) composed of isoechogenic solid-cystic nodules with macrocalcifications, particularly notable in Patient A. The histopathology report revealed that the thyroid gland was composed of numerous hypocellular nodules containing pink colloid. The hyperplastic nodules exhibited a vesicular structure with focal areas of papillary arrangement, characterized by intrafollicular centripetal growth. Some nodules demonstrated areas of nonspecific granulation, fibrosis, isolated calcifications, and a mixed-cellular inflammatory infiltrate, including foamy macrophages containing hemosiderin. The remaining thyroid parenchyma was mildly congested (**Figure 3**).

##### 3.9.1.4 Thyroid follicular adenoma

The US assessment revealed large, oval to round, solid nodules with mixed hypo-, hyper-, and isoechoic patterns, surrounded by a hypoechoic halo and displaying intranodular vascularization. Histological examination revealed encapsulated nodules with a capsule that was focally thickened and irregular, but without evidence of capsular invasion. The follicles within the nodules were tightly packed, while adjacent thyroid follicles were constricted, larger (containing more colloid), and elongated. The nuclei were enlarged, with nuclear clearing and frequent overlap (**Figure 4**).

##### 3.9.1.5 Oncocytic adenoma

US revealed well-demarcated, round to oval hyperechogenic nodules with small hypoechogenic foci and increased mixed-type vascularity. Histopathological examination showed densely packed eosinophilic cells, with the hypoechogenic foci corresponding to granular inflammatory tissue. Some cases exhibited advanced fibrosis or medium-sized vessels, consistent with the hyperperfusion observed in the US (particularly in cases A and C). The cells were pleomorphic, with enlarged nuclei and prominent nucleoli (**Figure 5**).

#### 3.9.2 Low risk/borderline tumors

##### 3.9.2.1 NIFTP

The US of NIFTP revealed oval to round nodules with regular margins. The capsule appeared as a hypoechoic rim, except in markedly hypoechoic nodules. The echogenicity of NIFTP cases was generally hypoechoic, with an US artifact of acoustic (posterior) enhancement visible below the nodules (**Figure 6**).

Histopathological examination of NIFTP revealed follicular structures ranging from microfollicles to macrofollicles. The nodule was round or oval, well-defined, with or without a fibrotic capsule, and without capsular or vascular invasion. The nuclei exhibited features of PTC, with a focal grade 3 nuclear score (**Table 4**).

##### 3.9.2.2 FT-UMP

In our study group, all patients had a nodule incidentally detected on US. US imaging revealed a small, round to oval, predominantly well-defined nodule with hypo- and hyperechoic areas. In some cases, the borders of the nodules were difficult to delineate. Vascularization within the nodules was mixed (**Figure 7**).

Histopathological examination revealed follicular structures ranging from micro- to macrofollicles. The nodules were round to oval, with uncertain foci of capsular invasion. There were no nuclear features indicative of PTC (**Table 4**).

##### 3.9.2.3 WDT-UMP

Ultrasound imaging revealed medium-sized, fairly well-defined hypoechoic nodules, sometimes with additional hyperechoic areas, appearing round to oval (**Figure 8**).

In one case, the nodule was found in the context of a multinodular goiter. Histopathological examination showed follicular structures, predominantly microfollicles. The nodule was oval and mostly well-defined, but with evidence of capsular and vascular invasion, and the nuclei displayed features of PTC (**Table 4**).

#### 3.9.3 Malignant tumors

##### 3.9.3.1 Papillary thyroid carcinoma

US imaging revealed an irregularly contoured, hypoechogenic nodule with increased mixed vascularization (central and peripheral) (**Figure 9**).

Histopathological examination revealed a mixture of follicles of varying sizes, composed of polymorphic cells with nuclei exhibiting “glassy” clearing and grooves, penetrating the capsule.

##### 3.9.3.2 Follicular thyroid carcinoma

US imaging showed large hypo- and hyperechogenic nodules, though the borders, while appearing well-defined, often had areas of uncertain demarcation with small hyperechogenic fragments (**Figure 9**).

Histopathology showed follicular structures ranging from micro- to macrofollicles with capsular and/or angio-invasion. The cells were pleomorphic, with large overlapping nuclei.

##### 3.9.3.3 Poorly differentiated thyroid carcinoma

PDTC manifested as large, heterogeneous, hypoechoic masses on ultrasound (**Figure 9**).

Histopathological analysis revealed features suggestive of neuroendocrine differentiation, including salt-and-pepper nuclei and trabecular structures.

## 4 Discussion and literature overview

In this study, we presented the most common etiologies of thyroid nodules observed in our center, integrating US findings with corresponding histopathological features and referencing the current literature. The primary aim of this part was to provide a comprehensive overview of the latest knowledge on relatively newly identified borderline nodules, which are rare in pediatric patients, positioning them in relation to both benign and malignant thyroid tumors. By offering this comparative context, we aim to clarify the distinguishing characteristics of these borderline lesions and support their effective differentiation from other thyroid pathologies in clinical practice.

### 4.1 Benign thyroid nodules

#### 4.1.1 Dyshormonogenetic goiter

DHG is the second most common cause of congenital hypothyroidism, accounting for 10%–15% of all cases, following thyroid dysgenesis (25, 26). The incidence of DHG is approximately 1 in 30,000 to 50,000 live births, occurring twice as frequently in female patients (25, 26). Thyroid enlargement in DHG is due to defects in thyroid hormone synthesis (25, 26). Pathogenic variants in genes involved in this process include those responsible for thyroglobulin synthesis (TG), iodide transport across the basal (NIS/SLC5A5) and apical (PDS/SLC26A4) membranes of the follicular cell, hydrogen peroxide generation (DUOX2 and DUOXA2), iodide organification (TPO), coupling of mono- and diiodotyrosine (TPO), and the proteolytic breakdown of thyroglobulin and iodide recycling (IYD/DEHAL1) (25, 26). A deficiency in circulating thyroid hormones leads to the activation of TSH secretion, which, in turn, causes hyperplasia of the defective thyroid gland (27). This can result in the development of tumors, such as FTC and PTC, and, less commonly, follicular adenoma (27). To date, approximately 30 cases of DHG associated with thyroid carcinoma have been reported, including four pediatric cases involving a newborn and children aged 6, 14, and 17 years (26, 28–30).

In both of our patients with DHG, US imaging revealed an enlarged thyroid with solid hypoechoic nodules, consistent with previous reports (31). FNAB yielded results of V and III, respectively, indicating a ROM of approximately 28% (9, 12). As a result, uneventful lobectomies were performed at ages 8 and 18 years. The average age of surgery due to nodular goiter in DHG, as reported in the literature, is 16 years (32).

Histologically, DHG is characterized by markedly hypercellular nodules, with predominant patterns including solid, microfollicular, macrofollicular, trabecular, and insular nodules (26). Additional features include papillary hyperplasia, absence of colloid, frequent internodular bizarre cells, and bridging fibrosis, as reviewed by Bychkov et al. (26).

#### 4.1.2 Thyroid follicular nodular disease (multinodular goiter)

The term “follicular nodular disease (FND)” was introduced in the WHO 2022 Classification to describe multifocal hyperplastic or neoplastic lesions occurring in the clinical context of MNG (2). TFND is the most common thyroid gland disorder, detected in 60% of benign tumors in this study. Ninety percent of affected patients are women (33). Autopsy reports estimate the prevalence of TFND at 10% to 40% (33). The ROM in TFND is between 3% and 5% (33, 34). Most patients with TFND are asymptomatic and euthyroid, as also observed in this study. Globally, iodine deficiency is the leading cause of TFND, while in Western countries, AIT is more prevalent (33). In the pediatric population, TFND more commonly develops during adolescence (33). The genetic basis of the disease, especially in pediatrics, includes tumor-predisposing syndromes such as familial adenomatous polyposis, PTEN hamartoma tumor syndrome (Cowden syndrome), Werner syndrome, Carney complex, Pendred syndrome, McCune–Albright syndrome, and DICER1 syndrome (9, 12, 13, 35–40).

As reviewed by Satturwar et al., TFND nodules can display a variety of US features, including isoechoic or hyperechoic nodules with a hypoechoic halo, a sponge-like or honeycomb pattern, anechoic areas containing colloid, and internal calcifications (33).

Satturwar et al. reported that TFND nodules may present a wide range of histological patterns, from colloid-rich and microfollicular to hypercellular and microfollicular (33). Secondary changes such as fresh or old hemorrhage, follicular rupture with a granulomatous response, fibrosis, calcification, and even osseous metaplasia may also be observed (33). Some cystically dilated follicles may exhibit papillary projections (Sanderson polsters) that mimic papillary carcinoma, although they lack the nuclear features characteristic of papillary carcinoma (33, 41–43).

#### 4.1.3 TFND in DICER1 syndrome

The DICER1 gene, located on chromosome 14q32.13, plays an important role in normal thyroid gland development (44–47). Multiple thyroid abnormalities have been identified in DICER1 syndrome, in addition to other non-thyroidal neoplasms (44–47). In 2011, Rio et al. reported that individuals carrying a germline pathogenic variant of DICER1 have an increased predisposition to developing TFND/MNG, with a 16- to 24-fold higher risk of TC compared to the general population (46, 47). Somatic DICER1 pathogenic variants are associated with thyroblastoma and childhood-onset PDTC, whereas germline variants are linked to TFND, follicular adenoma with papillary architecture, PTC, and FTC (47).

In our study, US revealed MNG composed of isoechogenic solid-cystic nodules with macrocalcifications, particularly notable in Patient A as previously reported (46, 47).

As reviewed by Riascos et al., TFND DICER+ is histologically characterized by the presence of multiple bilateral nodules showing follicular proliferations (47). These nodules may present as adenomatous nodules, macrofollicular-pattern nodules, well-circumscribed adenomas, or nodules with intrafollicular centripetal papillary growth, similar to those observed in our study (47). This growth pattern is often referred to as papillary hyperplasia or papillary adenoma, but it lacks the nuclear features typical of PTC (48). In patients with multiple adenomatous nodules, Cowden syndrome should be excluded (49). The suspicion of DICER1-related pathogenesis should be heightened when variable involutional changes are observed in the non-nodular thyroid parenchyma, as noted in our study group (48).

#### 4.1.4 Thyroid follicular adenoma

TFA is a benign, encapsulated tumor characterized by thyroid follicular cell differentiation, without capsular or vascular invasion, and lacking the nuclear features of PTC (50).

The incidence in the general population is approximately 3%–5%, predominantly affecting adults, typically in the fifth to sixth decades of life, with a higher prevalence in female patients (50, 51). The etiology is usually sporadic, though it may occur following radiation exposure or as a result of iodine deficiency (50, 52). TFA can also be observed in the pediatric population, particularly in association with familial tumor syndromes such as PTEN syndrome, Carney complex, MEN1 syndrome (Wermer syndrome), and McCune–Albright syndrome (50, 53, 54). Most patients are euthyroid, though hyperthyroidism can occur in cases of hyperfunctioning adenomas, especially in McCune–Albright syndrome (50).

According to Agarwal et al., US in TFA typically shows solid or solid-cystic nodules with smooth, well-defined margins, homogeneous or heterogeneous echotexture, isoechoic or hypoechoic characteristics, and sometimes a peripheral hypoechoic halo (50, 55–57). Blood flow is either absent or low (50, 55–57). In our study, US assessment revealed large, oval to round, solid nodules with mixed hypo-, hyper-, and isoechoic patterns, surrounded by a hypoechoic halo and displaying intranodular vascularization.

As reviewed by Agarwal et al., histopathological evaluation of TFAs shows that they are architecturally and cytologically distinct from the surrounding gland, causing compressive changes in the adjacent thyroid tissue (50). They are encapsulated by a thin to moderately thick capsule (50). The nodule structure can vary, presenting as normofollicular, microfollicular, macrofollicular, or solid/trabecular (50). A focal papillary pattern may be seen in hyperfunctioning adenomas and follicular adenomas with papillary hyperplasia (50). The nuclear features of PTC are absent (14, 50).

#### 4.1.5 Oncocytic adenoma

Oncocytes are enlarged, polygonal to square-shaped epithelial cells with distinct cell borders and a voluminous, granular, eosinophilic cytoplasm, resulting from the accumulation of mitochondria (58). Oncocytic change can be observed in various benign conditions, such as AIT, TFND, or MNG, particularly in patients who have undergone head and neck radiotherapy, systemic chemotherapy, or in benign and malignant thyroid neoplasms (59).

Oncocytic tumors (OCTs), formerly known as Hürthle cell tumors, are rare follicular-derived thyroid neoplasms, accounting for less than 5% of all thyroid tumors (2, 60). OCTs can be classified as either adenomas or carcinomas (61). According to Bhattacharyya et al., OCTs are more common in women (68%) and typically occur in the sixth decade of life (62). While most OCTs are benign (OCA), up to 40% have been reported to be malignant [oncocytic cell carcinoma (OCC)] (61, 63).

To date, only three cases of OCA have been described in children (60, 61, 64). In our study, we presented four pediatric cases, including one patient who had received brain radiotherapy for acute lymphocytic leukemia.

As reported by Asa et al., an oncocytic nodule is diagnosed when more than 75% of the lesion is composed of oncocytes (59). Surgical histopathology remains the gold standard for confirming OCA, offering high diagnostic accuracy (64, 65). OCA typically presents unilaterally and is treated with hemithyroidectomy (64, 66). In contrast, OCC can be bilateral and more aggressive, necessitating total thyroidectomy (64, 66).

### 4.2 Low-risk/borderline tumors

#### 4.2.1 NIFTP

This type of borderline tumor was previously referred to as noninvasive EFVPTC but was reclassified as NIFTP based on a consensus study by Thompson et al., which demonstrated its indolent biological behavior, characterized by a lack of metastasis or recurrence (15).

NIFTPs are encapsulated or well-circumscribed solid nodules, measuring up to 8 cm, with a follicular growth pattern and nuclear features typical of PTC (67–69). NIFTP accounts for approximately 9.1% of all papillary TCs and is occasionally reported in the pediatric population (70–73).

The diagnosis of NIFTP requires a surgically excised specimen, with comprehensive tumor evaluation to exclude capsular invasion (14, 15, 70). NIFTP is considered a borderline RAS-lineage tumor, situated between follicular adenoma and follicular carcinoma or invasive EFVPTC (14, 15, 70). The disease course is indolent, with excellent long-term survival following surgical excision, and lobectomy or partial thyroidectomy is usually sufficient (14, 15, 70).

According to Yang et al., the echogenicity of NIFTP can vary, ranging from markedly hypoechoic to hypoechoic, isoechoic, or mixed hypoechoic and isoechoic with cystic changes (1). Ultrasonographic characteristics of NIFTP include a wider-than-taller shape, smooth borders, occurrence in multinodular glands, and the absence of calcifications, with perinodular and intranodular vascularization. NIFTPs are ultrasonographically similar to follicular adenoma and minimally invasive follicular carcinoma (74, 75).

The inclusion criteria for NIFTP diagnosis include major features such as encapsulation or clear demarcation, a follicular growth pattern with less than 1% papillae, and a nuclear score of 2 or 3, characterized by nuclear enlargement, crowding/overlapping, elongation, irregular contours, grooves, pseudoinclusions, and chromatin clearing (14, 15, 70). Exclusion criteria include any capsular or vascular invasion, true papillary structures exceeding 1% of the tumor volume, psammoma bodies, an infiltrative border, tumor necrosis, increased mitoses, and features of other PTC variants or oncocytic lesions (14, 15, 70). Additional exclusion criteria include the presence of *BRAF V600E* and telomerase reverse transcriptase (*TERT*) promoter pathogenic variants and distant metastasis (76).

#### 4.2.2 FT-UMP

Follicular thyroid tumor of uncertain malignant potential (FT-UMP) was first proposed by Williams et al. in 2000 and is defined as “an encapsulated or well-circumscribed tumor composed of well-differentiated follicular cells, lacking the nuclear features of PTC, with questionable capsular or vascular invasion” (77, 78).

Ito et al. investigated the clinical characteristics of 339 patients with FT-UMP and reported that five patients (1%) experienced distant recurrence during postoperative follow-up (79). Ito et al. concluded that while FT-UMP is generally an indolent disease, some patients may experience distant recurrence, indicating the need for continued follow-up (79). However, the optimal duration of postoperative surveillance remains unclear (79).

In our study group, US imaging revealed a small, round to oval, predominantly well-defined nodule with hypo- and hyperechoic areas. In some cases, the borders of the nodules were difficult to delineate, similar to the findings reported by Ito et al. (79). Vascularization within the nodules was mixed.

Histopathological examination revealed follicular structures ranging from micro- to macrofollicles. The nodules were round to oval, with uncertain foci of capsular invasion. There were no nuclear features indicative of PTC.

#### 4.2.3 WDT-UMP

Well-differentiated thyroid tumor of uncertain malignant potential (WDT-UMP) is a follicular neoplasm characterized by ambiguous nuclear features of PTC and questionable capsular or vascular invasion (80, 81). Most cases exhibit an indolent clinical course (77). The diagnosis is based on morphological criteria, as immunostaining is not considered reliable (77, 81). The terminology was proposed by Chernobyl pathologists to prevent unnecessary aggressive treatment (77, 81). Notably, in two children from our cohort, WDT-UMP developed following chemotherapy for ALL and after total body irradiation prior to bone marrow transplantation for chronic granulomatous disease. WDT-UMP typically presents as a well-circumscribed or encapsulated solid nodule with an excellent prognosis following lobectomy (80).

As reviewed by Wei, the differential diagnosis includes FT-UMP (a follicular neoplasm with equivocal vascular or capsular invasion but without the nuclear features of PTC) and NIFTP (a follicular neoplasm with nuclear features of PTC but without vascular or capsular invasion) (14, 80, 82).

### 4.3 Malignant tumors

#### 4.3.1 Papillary thyroid carcinoma

PTC represents over 90% of all TC cases in children (9, 12, 13). Recent data from Siegel et al. indicate that TC constitutes 12% of cancers in adolescents and 2% in children under 14 years of age (83). In the United States, TC ranks as the fourth most common cancer in adolescents and the seventh most common in children (83). According to the Polish National Cancer Registry, new cases of TC in individuals under 19 years account for 2.3% of all TC diagnoses (12, 13). Among solid tumors, TC is the second most frequent in girls and the eighth in boys (12, 13).

In pediatric PTC, the most prevalent genetic alterations include RET-PTC and NTRK fusions, while pathogenic variants in BRAF V600E and RAS occur less frequently compared to adults (84, 85). Differentiated thyroid carcinomas in infancy, as reviewed by Riascos et al., are strongly associated with germline DICER1 pathogenic variants or DICER1 syndrome (47). Moreover, the presence of PDTC or thyroblastoma should prompt consideration of somatic DICER1 pathogenic variants (47).

In our cohort, US imaging revealed an irregularly contoured, hypoechogenic nodule. US characteristics indicative of thyroid malignancy include solid composition (typically hypoechoic), irregular shape and margins, a taller-than-wide configuration, microcalcifications, predominant intranodular over peripheral vascularity, rapid growth progression, and cervical lymph node enlargement (13, 20). Histopathological examination (HE) revealed a mixture of follicles of varying sizes, composed of polymorphic cells with nuclei exhibiting “glassy” clearing and grooves, penetrating the capsule.

#### 4.3.2 Follicular thyroid carcinoma

FTC is characterized by follicular differentiation without the nuclear features of papillary carcinoma (86). FTC accounts for 6%–10% of all thyroid carcinomas (86). Clinically, FTC may develop from a preexisting adenoma. It does not typically metastasize via lymphatics but rather spreads hematogenously to the lungs, liver, bones, and brain (86). Iodine deficiency is a known risk factor. FTC is more common in female patients, comprising 75% of cases, and typically presents at an older age than papillary carcinoma, with a peak incidence between 40 and 60 years, and is rare in children (86). The etiology includes iodine deficiency, radiation exposure, and older age (86). Molecularly, FTC is associated with activation of the PI3K/AKT or RAS pathways; NRAS and HRAS mutations are present in 49% of cases, PAX8/PPARγ rearrangements in 36%, and PI3CA and PTEN mutations in 5%–10% (88–91).

As reviewed by Wei, US may reveal a solid hypoechoic nodule with a peripheral halo (indicative of a fibrous capsule); irregular or poorly defined margins may suggest malignancy (86).

According to Wei, histopathological evaluation of FTC typically reveals a trabecular or solid follicular pattern (micro-, normo-, or macrofollicular), without the nuclear features of PTC (86). Features include invasion of adjacent thyroid parenchyma, complete capsular penetration, or vascular invasion (either within or beyond the capsule) (86). The capsule is typically thickened and irregular, requiring full-thickness penetration for diagnosis (86). Vascular invasion is characterized by endothelial-covered tumor within or beyond the capsule, attached to the vessel wall or with thrombus formation. Additional findings may include nuclear atypia, focal spindle cell areas, mitotic figures, and the absence of necrosis, squamous metaplasia, psammoma bodies, or significant lymphatic invasion (86–89).

#### 4.3.3 Poorly differentiated thyroid carcinoma

PDTC is classified under “follicular-derived carcinomas, high-grade” in the 2022 WHO classification system (2). PDTCs are malignant neoplasms of follicular cells that demonstrate limited evidence of follicular cell differentiation (90). The clinical course of PDTC lies between that of well-differentiated thyroid carcinomas (such as papillary and follicular carcinoma) and anaplastic carcinoma (90–92). PDTC is rare in pediatric populations but more commonly affects older adults, typically between 55 and 63 years of age (93, 94). Iodine deficiency may serve as a risk factor, with some PDTCs developing *de novo* and others arising from the dedifferentiation of follicular or papillary carcinomas (95). The molecular pathogenesis of PDTC involves early events in thyroid carcinogenesis. Both *BRAF V600E*-like and *RAS-*like TCs in adults and probably also *DICER1* TCs in children can acquire additional genetic alterations—such as pathogenic variants in *TP53, TERT, CTNNB1*, and *AKT1* in adults—leading to progression toward high-grade malignancy (47, 96–99).

Clinically, PDTC often presents as a large, solitary thyroid mass, frequently associated with nodal and hematogenous metastases (96).

In our study, PDTC manifested as large, heterogeneous, hypoechoic masses on US, consistent with previous descriptions (97). As reviewed by Wei, the histologic diagnosis of PDTC, according to the Turin consensus criteria, is based on a solid/trabecular/insular growth pattern, absence of the nuclear features characteristic of papillary carcinoma, and the presence of at least one of the following: convoluted nuclei, three or more mitotic figures per 10 high-power fields, or evidence of necrosis (90).

## 5 Summary

This illustrative review evaluates US and histopathological features of pediatric thyroid nodules, based on cases from our tertiary thyroid center, highlighting current diagnostic challenges and approaches. Following the 2022 WHO Thyroid Tumor Classification Update, which introduced “borderline” tumor categories, treatment decisions in pediatric endocrinology have shifted toward more individualized surgical strategies, such as opting for lobectomy over total thyroidectomy in specific cases (2, 13). At our center, lobectomy patients are regularly monitored until they reach 18 years old, when they are transitioned to adult endocrine care. The optimal follow-up duration for borderline tumors, however, remains undetermined.

The 2022 European Thyroid Association guidelines underscore US as a primary tool for distinguishing benign from malignant pediatric nodules, though its sensitivity and specificity can vary significantly based on features like hypoechogenicity, calcifications, nodule shape, margin irregularity, and vascularity (13). Since most US scoring systems are derived from adult data, which does not always translate well to pediatric nodules, we apply EU-TIRADS-PL grade 5 criteria in our center, lowering the threshold diameter for FNAB from 10 to 5 mm in children (20, 21). The EU-TIRADS scales in pediatrics serve as an adjunct tool, with our clinical approach incorporating both transverse and longitudinal US views, composition analysis, and continuous monitoring of small lesions to guide FNAB decisions. Furthermore, specific patient factors—age, gender, thyroid function, and risk history, such as cancer or radiotherapy exposure—play crucial roles in management decisions.

Suspicious findings on US lead to FNAB, which remains minimally invasive, cost-effective, and highly sensitive (13, 100). Since 2015, Poland has utilized TBSRTC for categorizing cytology findings (101). A recent study by Kujdowicz et al. found that FNAB sensitivity for PTC detection was 86% in non-AIT patients but only 61.5% in AIT patients, underscoring the need for surgical intervention consideration in pediatric AIT cases with Bethesda III–VI cytology (102). This group also reported that FNAB, using TBSRTC, can identify malignancies in thyroid nodules as small as 3 mm in diameter (102). This finding underscores the system’s sensitivity, particularly in pediatric cases, where smaller nodules and distinct pathological features are common.

The reclassification of NIFTP has led to updates in TBSRTC’s malignancy risk estimates for indeterminate categories (15, 22). Pediatric nodules generally carry a higher malignancy risk than adult cases, highlighting the importance of adapted TBSRTC classification for children.

This review identified overlapping US features among benign, borderline, and malignant nodules, demonstrating the limitations of US as a standalone diagnostic tool. Common benign and borderline features included well-defined, oval, smooth-margined nodules, while malignant tumors more frequently exhibited marked hypoechogenicity, irregular shape and margins, and microcalcifications. Malignant nodules also grow rapidly, present extrathyroidal invasion, reinforcing histopathology’s role in confirming diagnosis post-lobectomy and guiding postoperative follow-up (24).

Unique to TFND DICER1+ nodules was a cystic-solid composition, with macrocalcifications, indicating a need for genetic consultation (36).

This study’s limitations include retrospective data collection from a single center and a small sample size, focusing on the most illustrative cases. However, this review adds insight by detailing pediatric low-risk tumor features and our approach to management. For borderline tumors, such as FT-UMP, comprehensive follow-up is crucial due to reports indicating a risk of distant recurrence in some cases (79). Close monitoring helps ensure early detection of any recurrence and guides timely intervention if necessary, particularly given the uncertain behavior of these lesions in pediatric populations.

Future studies may improve US-histopathological correlations and provide new insights into borderline tumor follow-up. Additionally, artificial intelligence (AI) could enhance US’s role in pediatric thyroid management by simplifying risk assessments and potentially offering more personalized diagnostic outcomes, though further validation is needed to align AI models with specific patient populations (103, 104).

### 5.1 Conclusion

Because of the considerable overlap in sonographic features among benign, borderline, and certain malignant thyroid lesions in children, US alone is insufficient for reliable risk stratification. This overlap necessitates more frequent referrals for FNAB in pediatric patients compared to adults. Future studies incorporating advanced imaging techniques like elastography, enhanced cytopathology, and AI-driven analytics may provide new diagnostic solutions, especially given the increasing number of children presenting with solid thyroid nodules.

## Data Availability

The raw data supporting the conclusions of this article will be made available by the authors, without undue reservation.
